# Modulating the unfolded protein response with ISRIB mitigates cisplatin ototoxicity

**DOI:** 10.1038/s41598-024-70561-w

**Published:** 2024-09-27

**Authors:** Jiang Li, Stephanie L. Rouse, Ian R. Matthews, Yesai Park, Yasmin Eltawil, Elliott H. Sherr, Dylan K. Chan

**Affiliations:** 1 Department of Neurology, UCSF, San Francisco, USA; 2 Department of Otolaryngology-Head and Neck Surgery, University of California, San Francisco (UCSF), 513 Parnassus Ave, Rm 719, San Francisco, CA 94143 USA; 3 Department of Neurobiology, Harvard Medical School, Boston, USA; 4 Department of Pediatrics, Institute of Human Genetics, Weill Institute for Neurosciences, UCSF, San Francisco, USA

**Keywords:** Cisplatin, Ototoxicity, Unfolded protein response, Endoplasmic reticulum stress, Diseases, Cochlea, Neuroscience

## Abstract

Cisplatin is a commonly used chemotherapy agent with a nearly universal side effect of sensorineural hearing loss. The cellular mechanisms underlying cisplatin ototoxicity are poorly understood. Efforts in drug development to prevent or reverse cisplatin ototoxicity have largely focused on pathways of oxidative stress and apoptosis. An effective treatment for cisplatin ototoxicity, sodium thiosulfate (STS), while beneficial when used in standard risk hepatoblastoma, is associated with reduced survival in disseminated pediatric malignancy, highlighting the need for more specific drugs without potential tumor protective effects. The unfolded protein response (UPR) and endoplasmic reticulum (ER) stress pathways have been shown to be involved in the pathogenesis of noise-induced hearing loss and cochlear synaptopathy in vivo, and these pathways have been implicated broadly in cisplatin cytotoxicity. This study sought to determine whether the UPR can be targeted to prevent cisplatin ototoxicity. Neonatal cochlear cultures and HEK cells were exposed to cisplatin, and UPR marker gene expression and cell death measured. Treatment with ISRIB (Integrated Stress Response InhIBitor), a drug that activates eif2B and downregulates the pro-apoptotic PERK/CHOP pathway of the UPR, was tested for its ability to reduce apoptosis in HEK cells, hair-cell death in cochlear cultures, and hearing loss using an in vivo mouse model of cisplatin ototoxicity. Finally, to evaluate whether ISRIB might interfere with cisplatin chemoeffectiveness, we tested it in head and neck squamous cell carcinoma (HNSCC) cell-based assays of cisplatin cytotoxicity. Cisplatin exhibited a biphasic, non-linear dose–response of cell death and apoptosis that correlated with different patterns of UPR marker gene expression in HEK cells and cochlear cultures. ISRIB treatment protected against cisplatin-induced hearing loss and hair-cell death, but did not impact cisplatin’s cytotoxic effects on HNSCC cell viability, unlike STS. These findings demonstrate that targeting the pro-apoptotic PERK/CHOP pathway with ISRIB can mitigate cisplatin ototoxicity without reducing anti-cancer cell effects, suggesting that this may be a viable strategy for drug development.

## Introduction

Cisplatin is an effective chemotherapeutic agent used to treat many malignant solid tumors, yet treatment is associated with ototoxicity. Irreversible sensorineural hearing loss affects 75–100% of patients treated with cisplatin, with young children more susceptible than adults^1–4^. Despite some efficacy for sodium thiosulfate (STS) in the treatment of cisplatin ototoxicity in non-metastatic hepatoblastoma in children^5,6^, significant concerns have been raised that STS reduces overall survival especially in metastatic hepatoblastoma, possibly due to interfering with cisplatin’s anti-cancer effects^7^. There remains, therefore, a significant need for more specific pharmacological intervention to prevent ototoxicity while preserving cancer-cell cytotoxicity.

The cellular mechanisms underlying cisplatin ototoxicity are poorly understood. Upon exposure, cisplatin is taken up into hair cells principally through the copper transporter Ctr1^8^ and binds DNA, leading to generation of excessive reactive oxygen species (ROS), inflammation, and transcriptional arrest, ultimately causing apoptosis and necrosis of sensory hair cells as well as cells in the stria vascularis and spiral ganglion^9–13^. Efforts in drug development to prevent or reverse cisplatin ototoxicity have largely focused on pathways of oxidative stress and apoptosis, with some success^14^. There is extensive crosstalk between oxidative stress and endoplasmic reticulum (ER) stress^15^, as up to 25% of ROS are generated in the ER^16^, suggesting that ER dysfunction may be crucial to understanding cisplatin-induced cochlear cell apoptosis. ER stress occurs when improperly folded proteins accumulate in the ER lumen. In response, the unfolded protein response (UPR) is activated to either restore ER proteostasis, in the case of more transient stress, or induce apoptosis in severe stress^17^.

Cisplatin induces ER stress^18^; it directly interacts with proteins^19^ and promotes their unfolding^20^, and damage done to other organelles by cisplatin may act as an indirect inducer of ER stress^21^. Cisplatin ototoxicity is correlated with cytosolic protein synthesis inhibition^22^. In a rat model of cisplatin ototoxicity, BiP expression was broadly upregulated, indicating induction of ER stress. Enhancement of ER chaperone function and proteostasis with tauroursodexoycholic acid protected against cisplatin ototoxicity in vivo, indicating that the UPR is a viable target for therapy against cisplatin ototoxicity^23^. However, while ER stress has been implicated, the precise role of the UPR in cisplatin ototoxicity and cytotoxicity is not fully understood.

Our group implicated the UPR and ER stress in rapidly progressive genetic hearing loss due to absence of the novel deafness gene, TMTC4, as well as in noise induced hearing loss (NIHL) and cochlear synaptopathy^24,25^. We demonstrated that the UPR is activated within the cochlea of adult mice within two hours of noise exposure, and that treating mice with ISRIB (Integrated Stress Response InhiBitor), a small molecule activator of eIF2B that results in downregulation of CHOP and the pro-apoptotic arm of the UPR^26^, protects against NIHL and synapse loss.

Based on our findings implicating the UPR in genetic and noise-induced hearing loss, and the work of others implicating ER stress and the UPR in cisplatin ototoxicity^27,28^, we sought to further explore the role of the UPR in the pathogenesis and treatment of cisplatin-induced ototoxicity. In this study, we show that the UPR mediates cisplatin ototoxicity and demonstrate that ISRIB reduces cisplatin ototoxicity in vivo without affecting cisplatin’s cytotoxic effects on head-and-neck squamous cell carcinoma (HNSCC) cells in vitro. Our findings support the idea that the UPR is involved in the pathophysiology of cisplatin ototoxicity and may be an appealing target for therapeutic intervention.

## Results

### Non-linear cisplatin-induced cell death is correlated with UPR gene expression

**Fig. 1:**
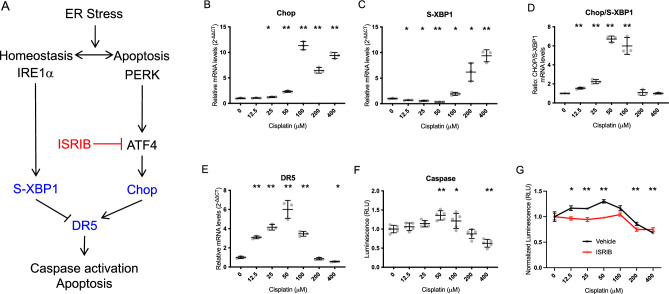
Non-linear cisplatin dose–response of UPR gene expression and apoptosis in HEK cells. (**A**) In the unfolded protein response (UPR), two major pathways, labeled by their initiating effectors (IRE1α﻿, and PERK) respond to accumulation of misfolded or unfolded proteins by facilitating homeostasis or, if the load is too great, apoptosis. The downstream effectors S-XBP1 and Chop are commonly used markers of the homeostatic (S-XBP1) and apoptotic (Chop) pathways. Depending on persistence and severity of stress, indicated by DR5, which integrates S-XBP1 and Chop signals, the UPR halts translation and allows for an adaptive response and return to proteostasis, or terminal response ending in apoptosis. ISRIB inhibits the PERK pathway by activating eiF2B and inhibiting ATF4. (**B**–**E**) UPR gene expression. Wild-type HEK cells were treated with multiple doses of cisplatin, and mRNA levels of Chop, S-XBP1, the ratio of Chop/S-XBP1, and DR5 were measured by qPCR and normalized to GAPDH by the 2^−ΔΔCT^ method. (**F**) Apoptosis. Caspase 3/7 activity was quantified using Caspase-Glo, demonstrating a non-linear cisplatin dose-dependence of HEK cells that matches DR5 expression. (**B**–**F**) Data are means ± SEM, with individual data shown as grey dots. *p < 0.05; **p < 0.001 by one-way ANOVA followed by Tukey multiple comparison test relative to control (0 μM cisplatin). Each graph is from a single experiment with 3 (**B**–**E**) or 5 (**F**) independent samples for each cisplatin dose. The experiment was repeated three times with identical results. (**G**) ISRIB treatment in cisplatin-treated cells reduces apoptosis. HEK cells treated for 40 h with cisplatin were additionally treated with 3 doses (at 0, 16, and 24 h) of 0.2 μM ISRIB (red), or vehicle (black), and Caspase 3/7 activity measured. Increasing cisplatin concentration induced an initial increase, then decrease, in apoptosis in the absence of ISRIB, consistent with the non-linear pattern of hair-cell death seen in cisplatin-treated cochlear cultures. At low cisplatin doses, ISRIB attenuated cisplatin-induced apoptosis and eliminated cisplatin dose-dependence; at high cisplatin doses, ISRIB had no effect. Data are means ± SEM, with individual values as pink or grey dots. *p < 0.05; **p < 0.01 compared to no drug (pairwise two-tailed t-test). N = 3 for each condition.

To explore the mechanistic relationship between cisplatin cytotoxicity and UPR gene expression, we first measured UPR marker gene expression in response to a broad range of cisplatin doses in HEK cells. UPR-mediated apoptosis and cell fate is regulated predominately through the actions of the IRE1α﻿ and PERK arms sending pro-homeostatic and pro-apoptotic signals, respectively (Fig. 1A). Death receptor 5 (DR5) integrates the opposing UPR signals of these two arms to control ER-stress-induced apoptosis: PERK/CHOP activity induces *DR5* transcription leading to formation of caspase 8 activating complex and ultimately apoptosis, whereas IRE1α/S-XBP1 promotes *DR5* mRNA decay, giving the cell time to restore homeostasis^29^. Thus, DR5 levels are a measure of the persistence of ER stress and likelihood of apoptosis, and are controlled by the opposing activities of the PERK/CHOP and IRE1α/S-XBP1 pathways.

As expected, cell viability decreased with increasing cisplatin dose (Supplemental Fig. S1). However, HEK cells exhibited a biphasic, bell-shaped response of UPR markers Chop, S-XBP1, and DR5 with increasing cisplatin dose, with a peak in expression of pro-apoptotic Chop and trough in expression of pro-homeostatic S-XBP1 at 50–100 µM cisplatin (Fig. 1B,C). The Chop/S-XBP1 ratio and DR5 expression were similarly elevated in this range, consistent with integration of the Chop and S-XBP1 pathways towards apoptosis (Fig. 1D,E). In accordance with this, we observed a peak in apoptosis, measured by caspase 3/7 activity, at 50 µM cisplatin (Fig. 1F). At higher cisplatin concentrations above 100 μM, where fewer cells remained viable (Supplemental Fig. S1), surviving cells exhibited high Chop levels but also high S-XBP1 levels, corresponding to lower Chop/S-XBP1 ratio, lower DR5, and less apoptosis.

Observation of this correlation of UPR gene expression and apoptosis across this non-linear, bi-phasic dose response led us to hypothesize that the UPR is causally involved in, and may be targeted to reduce, cisplatin-induced apoptosis. To test this, we treated cells with 0.2 μM ISRIB, an eIF2B activator that inhibits the PERK/CHOP arm of the UPR^26^ (Fig. 1A) and measured the effect on apoptosis across the same range of cisplatin doses. ISRIB reduced cisplatin-induced apoptosis at low cisplatin doses but had no effect at high cisplatin doses (Fig. 1G), suggesting that it may be an effective drug to reduce cisplatin ototoxicity.

### Cisplatin treatment induces the Unfolded Protein Response in the cochlea in vitro

**Fig. 2:**
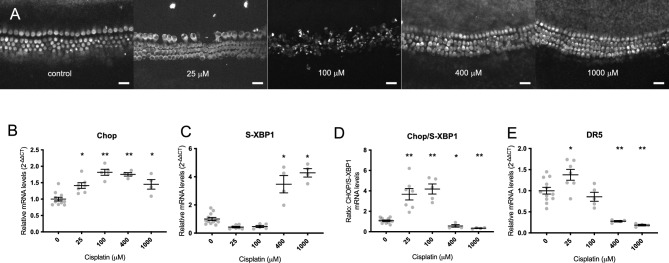
UPR modulation in cisplatin-treated cochleae. (**A**) Non-linear hair-cell death in response to cisplatin. Bell-shaped dose response in hair-cell death is observed in P3 WT C57BL/6J organotypic cochlear cultures treated with cisplatin at increasing doses (0–1000 μM) for 20 h then fixed and stained with anti-Myo7a to detect hair cells. At low concentrations of cisplatin, hair-cell death increases with increasing cisplatin concentration, but above 100 μM, cisplatin-treated cochleae had preserved hair cells. (**B**–**E**) Non-linear UPR gene expression in cochlear cultures quantified by qPCR after 6 h exposure to cisplatin. Chop (**B**), a marker of the pro-apoptotic arm of the UPR, and S-XBP1 (**C**), a pro-homeostatic UPR marker, displayed inverse non-linear dose-dependences. (**D**) The ratio of Chop/S-XBP1 demonstrated elevated balance towards pro-apoptotic Chop expression consistent with the pattern of hair-cell death seen in (**A**). The dose-dependency of this ratio was consistent with the expression pattern of DR5, a downstream marker that integrates the Chop and S-XBP1 signals and signals towards apoptosis. (**B**–**E**) Data are 2^−ΔΔCT^ measurements compared internally to GAPDH and normalized to control, non-treated cultures from the same experimental run, and are presented as means ± SEM, with individual data shown as grey dots. *p < 0.05; **p < 0.001 by one-way ANOVA followed by Tukey multiple comparison test relative to control (0 μM cisplatin). Scale bars 20 μm.

ER stress and the UPR have been broadly implicated in cytotoxicity induced by cisplatin treatment^18^. We quantified the effect of cisplatin on UPR markers in the cochlea. Explant cultures of P3-5 neonatal cochleae from wild-type C57BL/6J mice were treated with escalating concentrations of cisplatin for 20 h and then subjected to whole-mount immunohistochemistry against Myo7a, a hair-cell marker. Loss of inner and outer hair cells (IHCs and OHCs) occurred with increasing cisplatin dose up to 100 µM; with higher doses, hair cells were paradoxically preserved up to 1000 µM cisplatin (Fig. 2A).

Multiple groups have observed this bell-shaped dose–response of hair-cell death upon exposure of neonatal cochlear cultures to cisplatin: low cisplatin concentrations induce hair-cell death that increases with increasing cisplatin dose, whereas high cisplatin concentrations cause minimal hair-cell loss^22,27,28^. Based on our findings in HEK cells (Fig. 1), we hypothesized that the UPR may play a role in this response.

We performed qPCR on murine cochlear explant cultures exposed to the same escalating doses of cisplatin for 6 h, and measured Chop, S-XBP1, and DR5 expression. At this 6 h timepoint, all hair cells were still intact (Supplemental Fig. S2). Expression of these three markers exhibited a similar bell-shaped dose–response curve with inflection at 25–100 µM cisplatin (Fig. 2B–E). The highest expression level of the pro-apoptotic marker Chop was seen at a cisplatin dose of 100 µM, whereas the lowest expression level of the homeostatic marker S-XBP1 was seen at cisplatin doses of 25 and 100 µM. The ratio of Chop to S-XBP1, which reflects the balance between pro-apoptotic and pro-homeostatic arms of the UPR, demonstrated the same bell-shaped dose–response, and DR5, which integrates the two pathways and therefore reflects this Chop/S-XBP1 ratio, was maximally expressed at 25 µM cisplatin. The similarity between the unusual bell-shaped dose–response relationships of cisplatin with hair-cell death and pro-apoptotic UPR marker expression suggests that UPR activity may be involved in mediating cisplatin-induced hair-cell death.

**Fig. 3:**
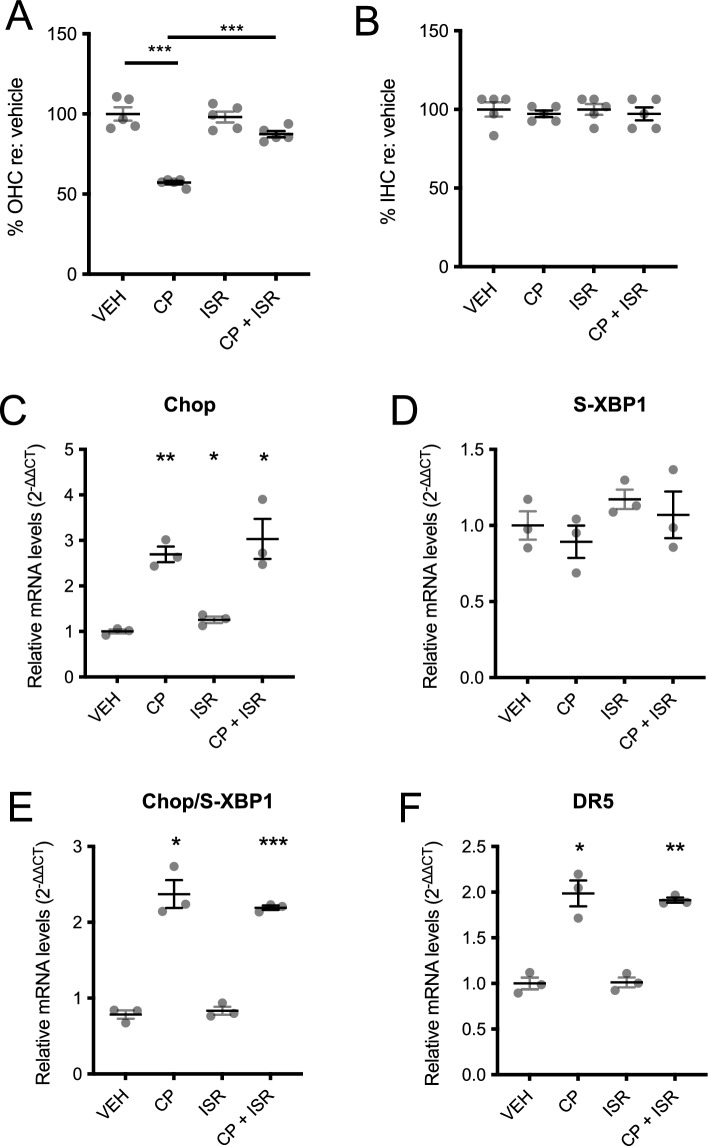
Effect of ISRIB in cisplatin-treated cochleae. Neonatal cochlear cultures were subjected to the following treatments: VEH, vehicle alone; CP, 100 µM cisplatin alone; ISR, 0.2 µM ISRIB alone; CP + ISR, 100 µM cisplatin together with 0.2 µM ISRIB. (**A**,**B**) Number of outer (OHC) and inner (IHC) hair cells 20 h after each of the indicated treatments was quantified, and the percentage of cells normalized against the number of cells in the vehicle condition. (**C**–**F**) Expression of Chop (**C**), S-XBP1 (**D**) and DR5 (**F**), and ratio of Chop/S-XBP1 (**E**) were quantified by qPCR after 6 h exposure. (**A**,**B**) Data are presented as means ± SEM, with individual data shown as grey dots. (**C**–**F**) Data are 2^−ΔΔCT^ measurements compared internally to GAPDH and normalized to control, non-treated cultures from the same experimental run, and are presented as means ± SEM, with individual data shown as grey dots. *p < 0.05; **p < 0.001; ***p < 0.0001 for two-tailed t-test on pairwise comparisons relative to control (VEH), or as indicated by black bars. All other pairwise comparisons were not significant.

We then tested the effect of ISRIB in cochlear cultures treated with cisplatin. After 20 h of treatment with 100 µM cisplatin, loss of OHCs, but not IHCs, was observed compared to cochleae treated either with vehicle or with 0.2 µM ISRIB alone (Fig. 3A,B). ISRIB, when used together with cisplatin, significantly reduced OHC loss (Fig. 3A), consistent with its effect in HEK cells. qPCR to measure Chop, S-XBP1, and DR5 expression in whole neonatal cochlear cultures after 6 h drug treatment showed an increase in Chop and DR5 expression, as well as Chop/S-XBP1 ratio, in cultures treated with 100 µM cisplatin. Though ISRIB co-treatment protected against cisplatin-induced OHC death (Fig. 3A), it had no effect on mRNA expression in whole cochleae (Fig. 3C–F).

### ISRIB protects against cisplatin-induced hearing loss and hair cell loss in vivo

**Fig. 4:**
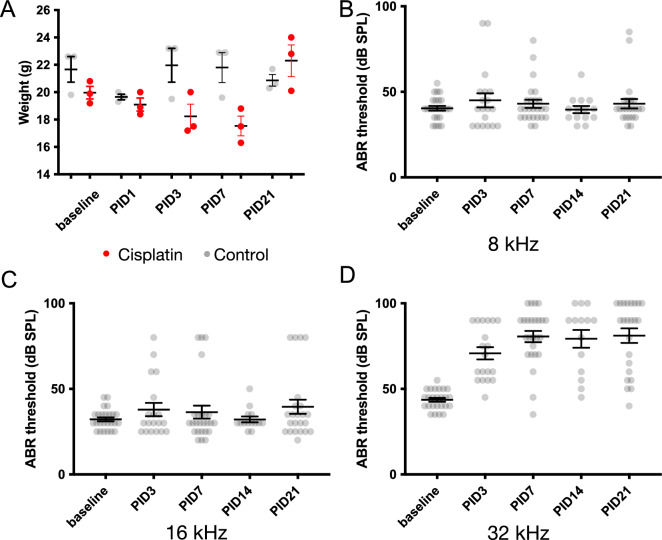
Balb/cJ model of murine cisplatin ototoxicity. A single intraperitoneal dose of 5.5 mg/kg cisplatin (CP) or saline was administered to 8-week-old female Balb/cJ mice. (**A**) Compared to control mice (gray, N = 3), weight decreased slightly in the CP group (red, N = 3) but returned to baseline by post-injection-day (PID)21. No mortality occurred. (**B**–**D**) ABR thresholds to 8, 16, and 32 kHz pure-tone pips were recorded at PID3, 7, 14, and 21 after treatment with 5.5 mg/kg cisplatin. Data shown are means ± SEM, with individual data shown in gray dots.

These in vitro experiments suggest that cisplatin may induce hair-cell death via ER stress and induction of the UPR, and that ISRIB, an eIF2B activator, is effective at reducing cisplatin-induced apoptosis and hair-cell death. Based on previous work suggesting that targeting ER stress can reduce cisplatin ototoxicity^23^ and our finding that ISRIB protects against ER stress and NIHL^24^, we hypothesized that ISRIB would also be effective at mitigating cisplatin ototoxicity. To test this, we used a model of cisplatin ototoxicity in Balb/cJ mice, a strain that has been shown to have low mortality with cisplatin treatment^30^. A single intraperitoneal (IP) dose of 5.5 mg/kg cisplatin administered to 8-week-old female wild-type Balb/cJ mice caused reproducible, isolated 32-kHz ABR threshold elevation at post-injection-day (PID) 21, with minimal morbidity and no mortality (Fig. 4). Cisplatin-treated mice experienced a transient loss of 10% body weight at PID7, with full recovery to baseline by PID21, with no mortality observed (N = 23), suggesting no overall impact on Balb/cJ morbidity.

**Fig. 5:**
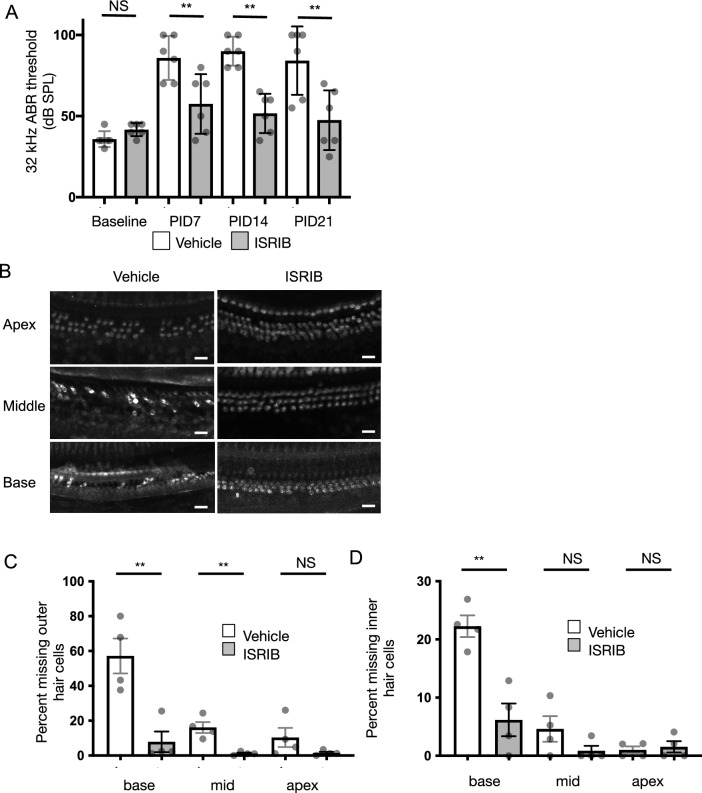
ISRIB protects against cisplatin-induced hearing and hair-cell loss. 8-week-old Balb/cJ mice were treated with a single intraperitoneal dose of 5.5 mg/kg cisplatin, and either 10 mg/kg ISRIB or vehicle. ABR thresholds to pure-tone pips were measured 7, 14, and 21 days after cisplatin treatment. After 21 days, mice were euthanized and temporal bones harvested. (**A**) 32-kHz ABR threshold measurements demonstrated significant protection against cisplatin-induced hearing loss in ISRIB-treated animals (gray) compared to controls (white). (**B**) Cochlear whole mounts from the apical, middle, and basal turns were stained with Myo7a and visualized by confocal microscopy, demonstrating significant hair-cell loss in cisplatin + vehicle treated animals (left), worse in the middle and basal turns. Cochleae from animals treated with ISRIB had better hair-cell preservation after cisplatin exposure. (**C**,**D**) Outer (**C**) and inner (**D**) hair cells were quantified as the percentage of missing cells in a fixed-length segment from the cochlear basal, middle, or apical turns from mice 21 days after treatment with cisplatin and either ISRIB (gray, N = 4) or vehicle (white, N = 4). Data shown are means ± 95% CI, with individual data shown in grey dots. **p < 0.01 by two-tailed t-test; *NS* not significant. Scale bars 20 μm.

10 mg/kg IP ISRIB was administered concurrent with cisplatin and serial ABR performed at PID7, 14, and 21. Cisplatin-induced 32 kHz ABR threshold elevation was reduced in ISRIB-treated mice compared with vehicle-treated controls at all 3 timepoints (Fig. 5A, p < 0.01). 21 days after cisplatin injection (PID21), ISRIB-treated animals had a mean 32-kHz ABR threshold of 47.5 ± 18.4 (mean ± SD, N = 6), compared with 84.2 ± 21.1 dB SPL for vehicle-treated controls, a statistically significant difference (p < 0.01, unpaired two-tailed t test). In fact, ISRIB-treated animals exhibited no significant elevation in 32-kHz ABR thresholds after cisplatin treatment (41.7 ± 4.1 dB SPL at baseline compared with 47.5 ± 18.4 dB SPL at PID21, p = 0.47). Overall, ISRIB treatment was associated with a 88% reduction in cisplatin-induced threshold shift compared to vehicle.

To evaluate further for ototoxicity, whole-mount immunohistochemistry was performed after 21 days of cisplatin exposure to quantify OHC and IHC loss. Whereas 57% ± 10% of basal-turn OHCs and 22% ± 2% of IHCs (mean ± SEM, N = 4) were lost in mice treated with cisplatin and vehicle, mice co-treated with ISRIB and cisplatin exhibited significantly less OHC and IHC loss (7.9% ± 5.9% and 6.2% ± 5.2%; p < 0.01 compared to vehicle by two-tailed t-test, respectively; Fig. 5B–D). These results demonstrate that ISRIB co-treatment protects against cisplatin-induced hearing loss and hair-cell death in this in vivo model of cisplatin ototoxicity.

### ISRIB does not affect the cytotoxicity of cisplatin against cancer cells in vitro

**Fig. 6:**
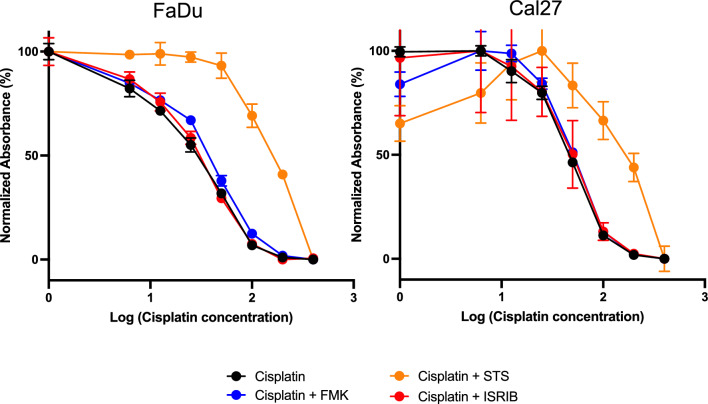
ISRIB does not interfere with cisplatin cytotoxicity against cancer cells. Viability of FaDu (left) and Cal27 (right) cells, head and neck squamous-cell carcinoma (HNSCC) cell lines, was measured in the presence of escalating doses of cisplatin alone (black) or in combination with 20 μM z-VAD-FMK (blue), an apoptosis inhibitor, 3 mM STS (orange), or 0.2 μM ISRIB (red). Data shown are means ± SEM (N = 3 for each condition).

A principal concern for any drug treatment for ototoxicity is potential interference with the anti-tumor effects of cisplatin. To test this, we used the FaDu and Cal27 head and neck squamous-cell carcinoma (HNSCC) cell lines (gifts of D.J.)^31^. Cisplatin treatment reduced FaDu cell viability, with an IC_50_ of 25.6 μM (R^2^ = 0.98). Co-treatment with 20 μM z-VAD-FMK, a caspase inhibitor known to inhibit cisplatin-induced hair-cell apoptosis^32^ as well as cisplatin chemotoxicity in cancer cells^33^, significantly increased the IC_50_ of cisplatin (33.3 μM (R^2^ = 0.98); IC_50_ shift ratio re: control = 1.32 (95% CI 1.19–1.48), Fig. 6). Similarly, co-treatment with 3 mM STS, the only effective drug for cisplatin ototoxicity humans that also has been shown to be associated with worse survival in disseminated hepatoblastoma^7^, significantly increased the IC_50_ of cisplatin (151.6 μM (R^2^ = 0.97); IC_50_ shift = 10.55 (9.17–12.21)). In contrast, co-treatment with 0.2 μM ISRIB, which prevented cisplatin-induced apoptosis in HEK cells (Fig. 1A), did not affect the IC_50_ of cisplatin (27.7 μM (R^2^ = 0.98); IC_50_ shift = 1.01 (95% CI 0.90–1.14)) in FaDu cells. Similar findings were observed using Cal27 cells, with cisplatin alone showing an IC_50_ of 22.6 μM (R^2^ = 0.94), STS shifting IC_50_ significantly to 72.5 μM (R^2^ = 0.76; IC_50_ shift = 11.61 (8.46–16.90)), and ISRIB having no effect, with an IC_50_ of 23.9 μM (R^2^ = 0.88; IC_50_ shift = 0.99 (0.77–1.28)). These findings demonstrate that ISRIB does not reduce the chemo-efficacy of cisplatin in two HNSCC cell lines in vitro.

## Discussion

Cisplatin is a frequently used chemotherapeutic agent with the potentially dose-limiting and life altering side effect of sensorineural hearing loss. However, the cellular mechanism underlying cisplatin ototoxicity is poorly understood. Here, we present evidence suggesting that the UPR may play an important role in mediating cisplatin-induced ototoxicity. Using a novel mouse model of cisplatin ototoxicity that produces consistent high-frequency hearing threshold elevation with minimal morbidity, we additionally show that modulation of the UPR with ISRIB protects against cisplatin-induced hearing loss and hair-cell death. Unlike the pan-caspase inhibitor z-VAD-fmk and the cisplatin ototoxicity drug STS, ISRIB does not impair the cytotoxicity of cisplatin in HNSCC cell lines. These findings suggest that targeting eIF2b using ISRIB may be a promising strategy to treat cisplatin ototoxicity without interfering with cisplatin chemo-efficacy.

### Non-linear dose–response of cisplatin and hair-cell death

We observed a bell-shaped dose response of hair-cell death upon exposure of neonatal cochlear cultures to cisplatin: low cisplatin concentrations induce hair-cell death that increases with increasing cisplatin dose, whereas high cisplatin concentrations give rise to little or no hair-cell loss. This phenomenon has been observed previously^27,28^, but the mechanism is unknown. One possibility is that cisplatin uptake may be altered at high cisplatin concentrations^27^, perhaps due to colloidal drug formulation at high concentrations^34^. Other studies have suggested that this phenomenon is an artifact of retention of hair cell “corpses” that retain hair-cell markers^35,36^. However, more recent studies suggest that even at high concentrations, cisplatin remains active and hair cells remain alive: 500 μm cisplatin treatment caused arrest of protein synthesis, but subsequent washout permitted recovery of protein synthesis and hair-cell survival^22^.

We observed in HEK cells and cochlear explant cultures that expression of Chop, S-XBP1, and DR5, markers of UPR activation specifically in the apoptotic, homeostatic, and integrated pathways, respectively, all displayed similar, unusual bell-shaped dose–response curves. This correlation suggests that the UPR plays a mechanistic role in cisplatin ototoxicity. The increase in S-XBP1 expression at high cisplatin concentrations, in particular, suggests that this protective response may underlie the reduction in hair-cell loss at these high concentrations. ISRIB, an eIF2B activator that suppresses the pro-apoptotic activity of the UPR, protected against cisplatin-induced apoptosis in the lower, physiologically relevant range of cisplatin doses, further supporting a mechanistic role of the UPR in cisplatin ototoxicity. When applied together with 100 μM cisplatin in cochlear cultures, ISRIB prevented death of OHCs, further supporting its ability to protect against ototoxicity.

Identification of different control pathways for cisplatin-induced cytotoxicity in different cell types could be of significant value in development of drugs to protect against cisplatin ototoxicity while retaining its cytotoxic properties for cancer chemotherapy. Studies in multiple cancer cell types have suggested that the homeostatic arm of the UPR plays a central role in chemoresistance^37–39^. In vitro, cisplatin affects cancer cells and hair cells differently: whereby cochlear hair cells exhibit the non-linear, bell-shaped dose response, cancer cell lines such as head and neck squamous cell carcinoma exhibit a typical monotonic dose–response^40^. It is possible that leveraging different dose–response characteristics relating to the UPR could lead to the development of UPR-targeting drugs that protect the cochlea while retaining anti-tumor activity. Indeed, we found that ISRIB, which specifically targets eIF2B, did not alter cisplatin’s cytotoxic effect on two HNSCC cell lines, providing strong initial evidence supporting this strategy. Multiple additional small molecule drugs have been developed to target different points in the homeostatic and apoptotic arms of the UPR. These can be used in vitro and in vivo to further test the hypothesis that the UPR is a control mechanism and target for treatment of cisplatin ototoxicity.

### ISRIB protects against cisplatin ototoxicity

The UPR is a potential target pathway for treatment of cisplatin ototoxicity. For this study, we refined a single-dose mouse model of acute cisplatin ototoxicity. Murine models of cisplatin ototoxicity are often complicated by high rates of mortality and a narrow ototoxic window compared with guinea pig and rat models^41^. Prior studies have suggested that Balb/cJ mice have increased susceptibility to cisplatin ototoxicity and reduced mortality compared with CBA/CaJ and C57BL/6J strains^30,42^. Owing to its selective high-frequency hearing loss, simplicity, and minimal morbidity, the Balb/cJ strain may be useful for more rapid evaluation and screening of candidate drugs, which can subsequently be tested in a more clinically relevant chronic administration paradigm.

We found that a single IP dose of ISRIB, administered concurrently with cisplatin, significantly reduced cisplatin-induced high-frequency hearing loss by 88%, with near-complete protection of cochlear IHCs and OHCs, particularly in the high-frequency basal turn, where the majority of damage occurred (Fig. 5). This corroborated our findings in cochlear cultures (Fig. 3A,B). ISRIB, a potent activator of eiF2B and thereby UPR-mediated apoptosis, has been found to effectively modulate the UPR in many disease models, including neurodegeneration^43^, memory loss^44^, and pulmonary fibrosis^45^. We found that ISRIB effectively protects against NIHL^24^ and cochlear synaptopathy^25^. The current findings suggest that the UPR may be a common mechanism in ototoxicity relating to noise and cisplatin. The efficacy of ISRIB corroborates prior findings: tauroursodeoxycholic acid, a non-specific modulator of the UPR, was effective at mitigating cisplatin ototoxicity in vivo^46,47^, and salubrinal, which targets eIF2a and, like ISRIB, modulates the PERK/CHOP pathway, protects against cisplatin toxicity in vitro^48^. Taken together, these findings suggest that targeting the UPR may be a valuable broad strategy to treat acquired hearing loss.

A critical consideration for any potential drug targeting cisplatin ototoxicity is potential interference with cisplatin’s chemoeffectiveness. Indeed, STS, the only currently FDA-approved drug that was shown to be effective in treating cisplatin ototoxicity^5^, was also found to be associated with reduced survival in disseminated hepatoblastoma. We found that STS shifts the IC_50_ of cisplatin in a HNSCC cell line by over tenfold, suggesting that it may indeed interfere with cisplatin’s anti-tumor effects. ISRIB, however, when applied at the same concentration found to reduce CHOP expression^24^ and apoptosis (Fig. 1) in HEK cells, does not affect the IC_50_ for cisplatin in this cancer cell line (Fig. 6). This suggests that specific targeting of the PERK-CHOP pathway of the UPR may be a valuable therapeutic strategy to mitigate cisplatin ototoxicity without interfering with its anti-tumor properties.

### Limitations and future directions

This study demonstrates that the UPR is involved in cisplatin ototoxicity; however, several limitations exist. We did not attempt to resolve which cochlear cell types are involved in this response; thorough analysis of the timecourse of the UPR response to cisplatin in different cochlear cell types is beyond the scope of the current investigation. Indeed, though ISRIB protected against cisplatin-induced OHC loss in cochlear cultures (Fig. 3A,B), it did not prevent cisplatin-induced Chop and DR5 upregulation upon bulk qPCR of the cochlear tissue (Fig. 3C–F), suggesting that its effect may be limited to certain cell types that is obscured with bulk qPCR analysis. Further investigation is necessary to identify the UPR response and ISRIB efficacy in specific cochlear cell types.

The UPR is a highly conserved mechanism We found that HEK cells exhibited similar behavior to cochlear cultures, including a similar inflection point of the biphasic response, though at the highest concentrations HEK cell viability was significantly reduced and we were likely measuring the UPR response of the few surviving cells. A more significant limitation is that the doses in which the paradoxical response was seen (> 100 μM) are well above those thought to be clinically relevant. However, even if these high cisplatin doses are not clinically relevant, the mechanisms uncovered by these supratherapeutic conditions that lead to hair-cell protection may be relevant when considering drug development for cisplatin ototoxicity.

Despite these in vitro limitations, the importance of the UPR in cisplatin ototoxicity is most strongly illustrated by our finding that ISRIB, which inhibits the pro-apoptotic arm of the UPR, can robustly protect against cisplatin-induced high-frequency hearing loss and hair cell death in vivo. As has been seen in prior investigations of cisplatin ototoxicity^49^ as well as other in vivo damage models such as NIHL^50^, there was not a precise correlation between ABR changes and loss of inner or outer hair cells, suggesting some mismatch between functional and structural changes in this model. As seen previously in a mouse model of cisplatin ototoxicity^49^, we found that OHC damage was more severe than IHC damage, which may partially underlie this phenomenon; thorough investigation into the timeline of functional and structural changes in this and other cisplatin ototoxicity models is required. We used a single-dose acute cisplatin model in Balb/cJ mice, which were chosen owing to their low mortality and consistent hearing threshold shift with cisplatin treatment, compared with CBA/CaJ and C57Bl/6J strains^30^. However, this acute model is in contrast with the chronic model of cisplatin administration and ototoxicity that occurs clinically in humans and that has been established in CBA/CaJ mice^49^. Corroboration and expansion of our findings in a more clinically relevant chronic administration model of cisplatin ototoxicity would be necessary to thoroughly understand its therapeutic potential.

## Conclusion

Cisplatin ototoxicity is a significant challenge with incompletely understood pathophysiology. This study provides evidence for the involvement of the UPR in cisplatin ototoxicity and demonstrates that targeting the pro-apoptotic arm of the UPR with ISRIB mitigates cisplatin-induced hearing loss and hair-cell death in mice without affecting cisplatin chemo-efficacy in HSNCC cell lines. Further investigation of the role of the UPR in cisplatin ototoxicity, and the potential for targeting it for treatment, is warranted.

## Methods

### Sex as a biological variable

Our study examined female mice, because the cisplatin ototoxicity model we used, described below, had less variance in hearing outcomes in female compared with male mice in pilot studies. Therefore, using exclusively female mice in these initial studies reduced the number of animals required. Future studies will examine both female and male mice.

### In vivo mouse model of cisplatin-induced ototoxicity

As a single-dose model for acute cisplatin ototoxicity, 8-week-old female wild-type Balb/cJ mice (Jackson Laboratories, Strain #000651) were administered 5.5 mg/kg cisplatin (Sigma-Aldrich, 1134357 in normal saline) via intraperitoneal (IP) injection. To assess the effect of UPR modulation on cisplatin ototoxicity, we treated animals with ISRIB concurrent with cisplatin administration. Mice were injected intraperitoneally with 10 mg/kg ISRIB (Sigma, SML0843) or vehicle (50% DMSO, 50% PEG-400) immediately before cisplatin administration. Animals were administered 0.5 mL IP normal saline (NS) bolus for hydration concurrent with cisplatin and drug delivery, and subsequently on post injection days 1, 7, 14, and 21.

### Auditory testing

Hearing was tested in mice by measuring auditory brainstem response (ABR) thresholds in response to broadband tone pips at 8, 16, and 32 kHz in the sound field using a standard commercial system (RZ6, Tucker-Davis Technologies) in a soundproof chamber as described^24^. All ABR measurements were performed by an investigator blinded to drug treatment.

### Organotypic culture of the neonatal mouse cochlea

Organotypic cultures from wild-type P3-5 C57BL/6J mice (Harlan, 057) were established as described^24^. Briefly, temporal bones were extracted and both cochlear ducts isolated from P3 mice. Cochleae were opened and plated on glass coverslips coated with with Cell-Tak (Corning, 354240) with the apical surface of the epithelium facing up. Tectorial membrane was left intact and cochleae cultured overnight in DMEM supplemented with 10% FBS at 37 °C in 5% CO_2_. Cultures that were grossly intact and remained fully adherent to the coverslip without sign of contamination were used for experiments after 24 h in culture. Cisplatin was applied to cultures in media in increasing doses of cisplatin from 0 to 1000 μM for up to 20 h.

### UPR gene expression analysis in cell culture

Human embryonic kidney (HEK, purchased from UCSF Cell Culture core facility) cells were grown in DMEM supplemented with 10% FBS, at 37 °C in 5% CO_2_. To measure UPR marker gene expression in response to a broad range of cisplatin doses, HEK cells were treated with cisplatin (0–400 μM) for 40 h, and then total RNA was isolated using TRIzol Reagent (Invitrogen, Carlsbad, CA). 1 μg total RNA was used for first-strand cDNA synthesis using SuperScript IV VILO master mix (Invitrogen, Carlsbad, CA). Real-time PCR was performed by 7900HT FAST Real-time PCR system (Applied Biosystems, Foster City, CA) with a SYBR green I mix. The mRNA primers for UPR markers were as follows: *Chop*: CCACCACACCTGAAAGCAGAA (forward), AGGTGAAAGGCAGGGACTCA (reverse); *S-XBP1*: CTGAGTCCGAATCAGGTGCAG, GTCCATGGGAAGATGTTCTGG; *DR5*: TGCTGCTTGCTGTGCTACAGGCTGT, TTCTGACAGGTACTGGCCTGCTAG. Relative quantification by the 2^-ΔΔCT^ method was used for analysis as described previously^24^. To assess activation of Caspase 3 and 7, the Caspase-Glo® 3/7 assay was performed according to manufacturer’s instructions (Promega) on HEK cells after 40 h treatment with cisplatin. To test if UPR directly causes cisplatin-induced apoptosis, HEK cells treated for 40 h with 0–400 μM cisplatin were additionally treated with 3 doses (at 0, 16 and 24 h) of 0.2 μM ISRIB and the Caspase-Glo® 3/7 assay performed. To assess cell viability, a cell viability assay was performed (Promega CellTiter-Glo 2.0). Luminescence was recorded using a SynergyH4 microplate reader (Agilent Technologies, Hayward, CA) and IC_50_ curves analyzed (Prism 9, GraphPad).

### Immunohistochemistry

Evaluation and quantification of hair-cell loss was performed with whole-mount cochlear immunohistochemistry as previously described^24^. Briefly, cochlear explant cultures were treated as indicated and fixed in 4% paraformaldehyde. 8-week-old cisplatin-treated mice were euthanized 21 days after cisplatin treatment and temporal bones dissected, isolating the apical, middle, and basal cochlear turns. Specimens were incubated with rabbit anti-myosin7a antibody (a hair-cell-specific marker; 1:200 dilution in PBS; 25–6790, Proteus Biosciences) overnight at 4 °C. Cochleae were rinsed three times for 15 min with PBS and then incubated for 2 h with a goat anti-rabbit secondary antibody conjugated to Alexa Fluor 488 (1:1000 dilution in PBS; Life Technologies). Whole mounts were rinsed in PBS three times for 15 min and mounted on glass slides in VectaShield antifade mounting medium (Vector Laboratories). The hair-cell region of the organ of Corti was imaged on a Nikon A1R upright line-scanning confocal microscope as previously described. For 8-week-old cochleae, the imaging locations for the apical, middle, and basal turns corresponded approximately to the 8, 16, and 32-kHz regions of the cochlea^25^, and missing outer hair cells were counted from anti-Myosin7 immunostain in 200 μm cochlear segments and expressed as a percentage of missing outer hair cells.

### Cell viability in FaDu cells

FaDu and Cal27 cells (gift of Daniel Johnson), head and neck squamous cell carcinoma lines^31^, were cultured in 96-well plates at a density of 10,000 cells per well. Cells were allowed to adhere and grow for 24 h before treatment. Serial dilutions of cisplatin were prepared in cell culture medium at a range of concentrations from 0 to 200 µM, with 0.2 µM ISRIB, 20 µM z-VAD-FMK, or 3 mM STS. Plates were incubated for 48 h and cell viability assay performed (Promega CellTiter-Glo 2.0). Luminescence was recorded using a SynergyH4 microplate reader (Agilent Technologies, Hayward, CA) and IC_50_ curves analyzed (Prism 9, GraphPad).

### Statistics

For comparison between treatment groups for UPR gene expression, we used one-way ANOVA followed by Tukey multiple comparison tests. For pairwise comparison of ABR thresholds between groups of mice, we used unpaired two-tailed Student's t-test. Statistical analyses were performed with GraphPad Prism v9.

### Vertebrate studies approval and conduct

All experimental protocols were approved by the UCSF Institutional Animal Care and Use Committee (AN183160). All experiments were performed in accordance with these approved guidelines and regulations; specifically, euthanasia was performed by decapitation for neonatal mice and by CO_2_ euthanasia with cervical dislocation for adult mice. All methods are reported in accordance with ARRIVE guidelines.

## Supplementary Information

Supplementary Information 1.

Supplementary Figure 1.

Supplementary Figure 2.

Supplementary Legends.

## Data Availability

All data generated or analysed during this study are included in this published article and its supplementary information file.
