# Supplementary figures and images for: Modulating the unfolded protein response with ISRIB mitigates cisplatin ototoxicity
Source: Sci Rep. 2024 Sep 27;14:22382. doi: 10.1038/s41598-024-70561-w (PMC11437005; doi:10.1038/s41598-024-70561-w)

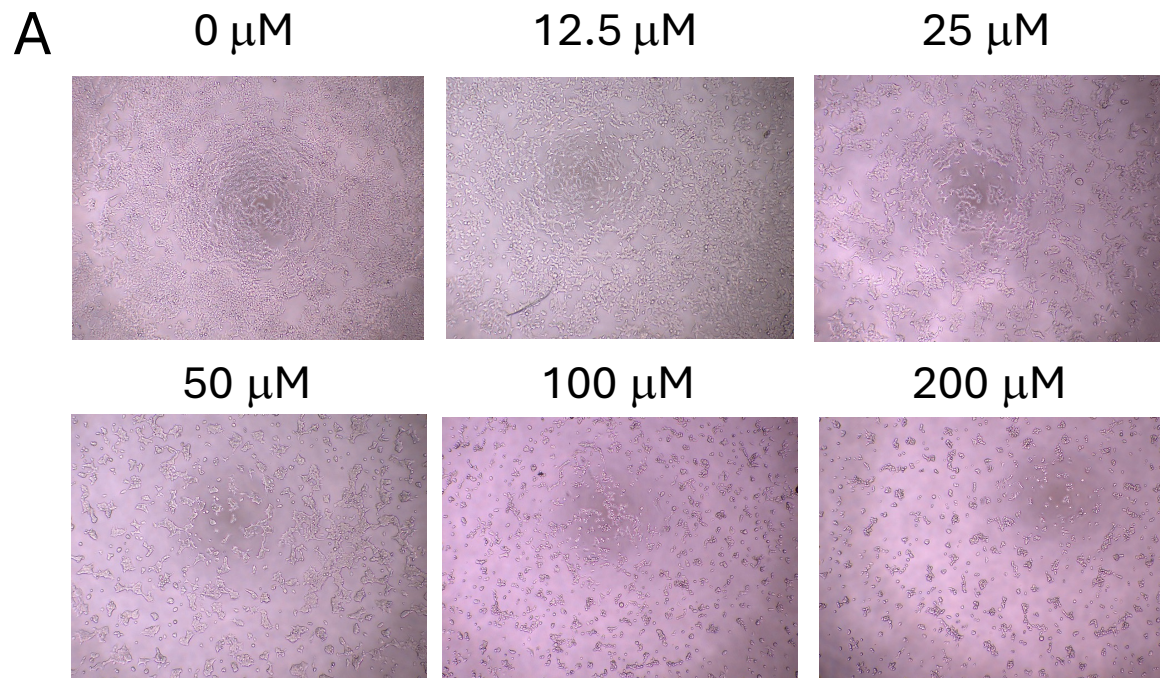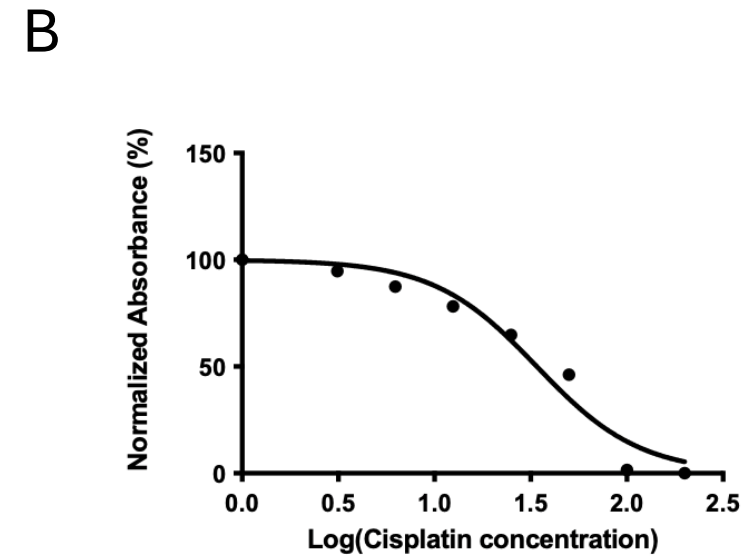

Supplement: Supplementary file 2 — Supplementary Figure 1. [file 41598_2024_70561_MOESM2_ESM.pdf]

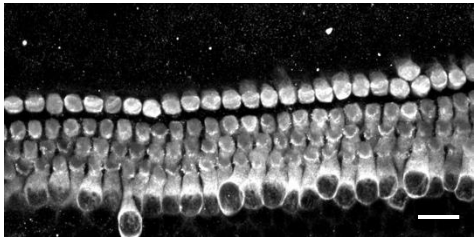

control

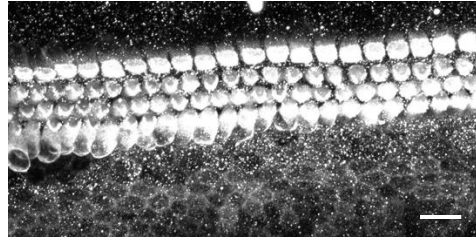

25  $\mu$ M

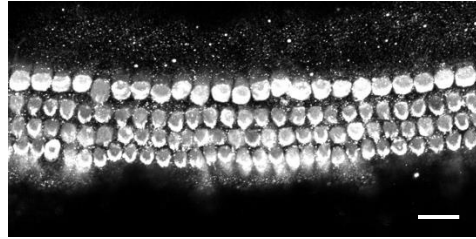

100  $\mu$ M

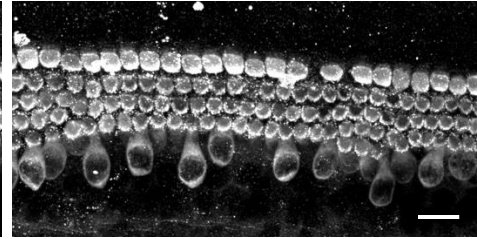

400  $\mu$ M

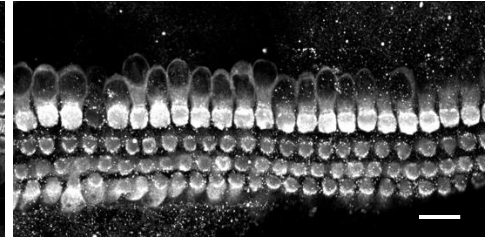

1000  $\mu$ M

Supplement: Supplementary file 3 — Supplementary Figure 2. [file 41598_2024_70561_MOESM3_ESM.pdf]
