# Supplementary material for: Modulating the unfolded protein response with ISRIB mitigates cisplatin ototoxicity
Source: Sci Rep. 2024 Sep 27;14:22382. doi: 10.1038/s41598-024-70561-w (PMC11437005; doi:10.1038/s41598-024-70561-w)
Supplement: Supplementary file 4 — Supplementary Legends. [file 41598_2024_70561_MOESM4_ESM.docx]

**Supplemental Figure 1. HEK cells treated with cisplatin.** Viability of HEK cells was measured in the presence of escalating doses of cisplatin. At 48h after treatment, cisplatin caused significant cell death, though some cells remained even at the highest cisplatin doses (**A**). IC_50_ for cisplatin was 34.1 μM (**B**). Data shown are means ± SEM (N=3 for each condition).

**Supplemental Figure 2. Cochlear cultures treated with cisplatin after 6h.** P3 WT C57BL/6J organotypic cochlear cultures were treated with cisplatin at increasing doses (0-1000 μM) for 6h then fixed and stained with anti-Myo7a antibody to detect hair cells.
